# A Narrative Review of Patellar Resurfacing Versus Non-resurfacing in Total Knee Arthroplasty

**DOI:** 10.7759/cureus.39362

**Published:** 2023-05-22

**Authors:** Sergiu Iordache, Mihai Costache, Adrian Cursaru, Bogdan Serban, Razvan Spiridonica, Mihnea Popa, Catalin Cirstoiu, Bogdan Cretu

**Affiliations:** 1 Orthopedics and Traumatology, University Emergency Hospital, Bucharest, ROU; 2 Orthopedics and Traumatology, Carol Davila University of Medicine and Pharmacy, Bucharest, ROU

**Keywords:** arthrosis, arthroplasty, knee, resurfacing, patella

## Abstract

The number of individuals who experience the symptoms of gonarthrosis rises proportionally as life expectancy rises and the population becomes more active. The purpose of total knee arthroplasty (TKA) is to lessen pain and restore knee function, and it has a high success rate. The restoration of patellar tracking in addition to the proper alignment of the femoral and tibial components contributes to the success of the arthroplasty and the patient's happiness. Replacement of the knee is not an easy process. One of the major objectives of total knee replacement is to achieve the proper rotation of the femoral components. A critical step that affects postoperative outcomes in total knee arthroplasty is the correct alignment of the femoral component.

The axial plane of the femoral component is to blame for flexion stability, knee joint kinematics, flexion alignment, and patellar tracking. The patella is the largest sesamoid bone in the human body, and its major role is to enhance the quadriceps' moment arm, which allows the knee to expand.

The distribution of patellofemoral compressive pressures during knee flexion and the centralization of the quadriceps muscles' multidirectional pull during extension are both critical functions of the patella. After primary knee arthroplasty, there are 8% more cases of anterior knee discomfort than there were before. Whether or not the patella was resurfaced, patients with primary TKA experience anterior knee discomfort.

Patella baja is caused by excessive joint line elevation, which causes persistent overload and discomfort. The design of the TKR might have an impact on postoperative patellofemoral problems. After TKR, patellofemoral maltracking and patellar dislocation are often caused by surgical mistakes.

## Introduction and background

Life expectancy is increasing, the population is becoming more active, and the number of people who are affected by gonarthrosis is increasing. By 2030, one in three adults in the United States is expected to suffer from osteoarthritis. Total knee arthroplasty (TKA) has high success rates, with the goal of decreasing pain and restoring knee function. Numerous studies have shown that the satisfaction rate of total knee arthroplasty patients is between 80% and 95%, and the survival rate of implants 10 years after the intervention is approximately 90% [1]. The main reasons for low satisfaction rates are prosthetic positioning deficiencies (especially at the level of femoral rotation), rotational mismatch between the femur and tibia, femoral-patellar maltracking, changes in the height of the joint line, instability, prosthetic loosening, polyethylene destruction, prosthetic fixation, and osteolysis, which may be secondary to infection or particle disease.

In medical applications, polymethylmethacrylate (PMMA), an acrylic polymer created by combining a powered MMA-styrene copolymer and a liquid methacrylate (MMA) monomer, is used for prosthetic fixation [2,3]. After combining the two parts, hardened PMMA is created as a result of the liquid monomer polymerizing around the prepolymerized powder particles. Due to the exothermic reaction, heat is produced during the process [2,4]. These are just some of the complications that can occur in the short, medium, or long term following knee arthroplasty. Some complications are directly related to the surgical technique used and can be avoided by following some operative standards. The vast majority of complications reside in a collection of error factors starting from the stage of preoperative evaluation where important aspects, particular to each patient, are not identified [5]. The knee joint is a complicated one with a high degree of mobility that permits not just sagittal and coronal plane movement but also internal femoral rotation movement relative to the tibia. One of the primary aims of total knee arthroplasty is to achieve proper femoral component rotation [5-8]. The femoral alignment during TKA is an important step that determines postoperative outcomes. The femoral component's axial plane location is crucial for flexion stability, knee joint kinematics, flexion alignment, and patellar tracking.

The restoration of patellar tracking in addition to the proper alignment of the femoral and tibial components contributes to the success of the arthroplasty and the patient's happiness. The primary function of the patella, the biggest sesamoid bone in the human body, is to increase the quadriceps' moment arm, which helps the knee extend [9]. The patella additionally centralizes the multidirectional pull of the four quadriceps muscles during extension and distributes patellofemoral compressive stresses during knee flexion [9].

The trochlear groove is just a circle that sits laterally to both the mechanical and anatomical axes. The trochlear axis produces a new distal femoral axis between the centers of the spheres attached to the trochlear facets [10].

According to the literature, a surgeon's choice may have a role in patella resurfacing [11]. The patella is resurfaced by certain surgeons always, never, or very sometimes [12].

When considering whether to resurface the patella, several factors are evaluated, including the patient's demographics (body mass index, mobility, etc.), the type of prosthesis being used, the position of the components, alignment, and correct soft tissue balance. Because of the position of the femoral and tibial components, the patella may engage at a different flexion angle. Anterior knee discomfort after primary knee arthroplasty has been found to occur in 8% of patients [13-16]. Patellofemoral maltracking is a major condition during TKA that affects the ultimate functional result and may increase the number of problems and revision surgery [17,18].

## Review

Patella replacement: rare, maybe not necessary at all (pros)

Patellar arthroplasty has long been performed in the United States and is considered standard practice in many regions. In many countries, however, the patella is very often unprosthetic. Knee replacement is not a simple procedure. The optimum surgical course of action for isolated patellofemoral arthritis is still up for debate. Total knee arthroplasty and patellofemoral replacement are the surgical options [19-22]. With some effectiveness, TKA has been used to treat isolated patellofemoral osteoarthritis; nonetheless, anterior knee discomfort persists [20-23].

There are several unfavorable consequences of prostheses, such as movement restrictions, lateral facial discomfort, late fracture, and avascular necrosis. Patellar component failures were documented in the research by Berend et al. [24] 4.2% of the time at a mean of just 2.6 years. They were followed up for an average of just 5.5 years, and very few needed surgery. All individuals with well-localized anterior knee discomfort were examined in a research recently carried out at the University of Washington to track the incidence of problems following total knee arthroplasty with and without patellar reconstruction.

Forty-seven cases of TKA patients with anterior knee discomfort were found during the four-year follow-up period, of which 36 had a prosthetic patella and 11 did not. Only two of the 11 patients with unrepaired patellas had further surgery, compared to eight of the 36 patients who received surgical repair of the prosthetic patella. Many more patients reported to the clinic with knee discomfort in cases where the patella was resurfaced, mostly because roughly the same number of complete knee arthroplasties were performed in the same clinic without patella resurfacing [25]. Patellar loosening, patellar fragmentation, avascular patellar necrosis, late stress fracture, lateral facet discomfort, oblique reattachment, and insufficient patellar thickness are pathologies that need to be treated following patellar replacement.

There was no change in the Oxford score, Short Form-12 (SF-12), or EuroQoL 5D (EQ-5D) at five years in a UK multicenter randomized clinical study that included nearly 1,700 knees from 34 locations and 116 surgeons nor was there a need for interventional further surgery for problems. The authors' statement that "No difference is seen in any score, and if there is a difference, it is too small a number to be clinically meaningful" was followed by their conclusion [26].

Regardless of whether the patella is artificial or not, the surgical method and component design play a significant role in the clinical result and existence of anterior knee discomfort in TKA. Rotational errors of the femoral or tibial component, implant maltracking, patellar fracture, aseptic loosening, and polyethylene wear are some of the causes of patellofemoral problems. Changes in the biomechanics of the knee, such as placing the femoral component in external rotation or the tibial component in internal rotation, might modify the kinematics of the patella in the trochlear groove. Changes in the tibiofemoral joint line can also be linked to changes in patellar kinematics, with elevations causing patellar problems such as inferior edge stress and patellar impingement. Patellar instability caused by patellar alta or patella baja syndrome is linked to anterior knee discomfort, patellar subluxation, or patellar dislocation occurrences [27,28].

The patellofemoral joint is essentially the only area where signs and symptoms of patellar replacement may be found, albeit the replacement of the patella is not essential in the great majority of patients [29].

Patella replacement: rare, maybe not necessary at all (cons)

The decision to resurface the patella was well-studied. While there are regional differences, the option for most surgeons in the United States is to resurface the patella. The data proves that this is the right choice.

Articular cartilage on metal has not proven to be a good long-term bearing surface. Lee et al. [30] also stated that the cartilage in the arthritic knee presents significant pathological abnormalities [31].

In certain situations, osteoarthritis impacts patellar thickness, and removing damaged bone results in a reduced patellar thickness, as well as the associated complications [32].

Patellar replacement has great lasting results and a low complication incidence. Anterior knee pain is a usual post-knee replacement complaint, and it is significantly more prevalent in TKA with a nonprosthetic patella [20,33]. Whenever the patella was not replaced, Pakos et al. [34] observed that there were greater reoperations and pain in the anterior area. In patients with unreconstructed patellar surfaces, Parvizi et al. [35] found a poor level of satisfaction. Studies show that nonprosthetic patellas require additional revisions.

Total knee prostheses with the highest revision rate are those with nonprosthetic patella according to the Swedish registry, with a 140% higher revision rate.

Patellar resurfacing could decrease the likelihood of reoperation and noise after surgery, as well as increase the Knee Society Score (KSS) and function score, according to Chen et al. [36]; however, it might not have an impact on outcomes such as anterior knee pain (AKP), range of motion (ROM), Oxford score, Knee Injury and Osteoarthritis Outcome Score (KOOS), visual analog scale (VAS), Feller score, patellar tilt, and patient satisfaction.

In addition, the second knee replacement surgery is often unsuccessful without pain relief.

**Figure 1:**
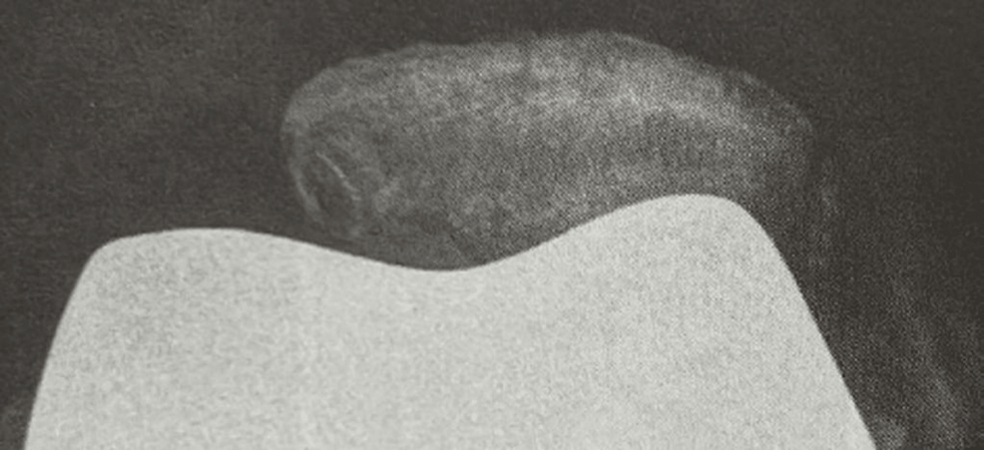
Postoperative skyline radiograph of a 62-year-old male suffering from hyper-compression syndrome of the patella leading to anterior knee pain

**Figure 2:**
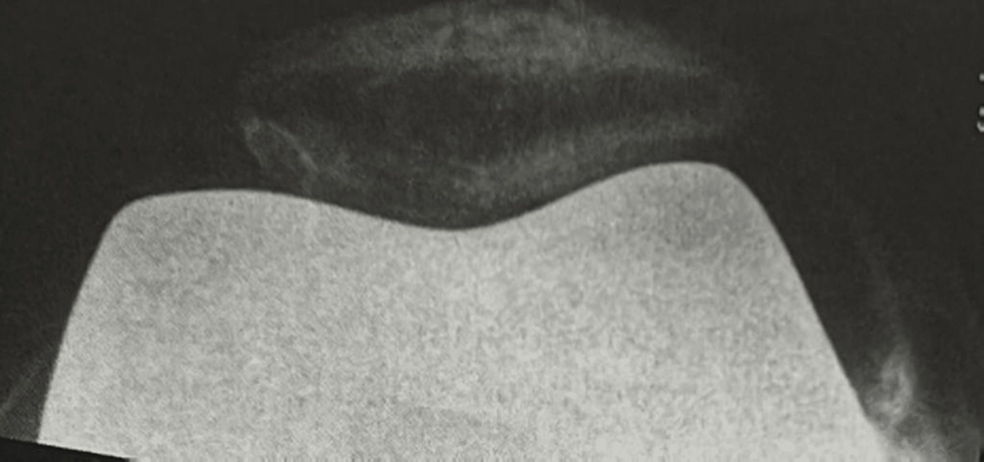
Partial lateral facetectomy with improved pain relief

**Figure 3:**
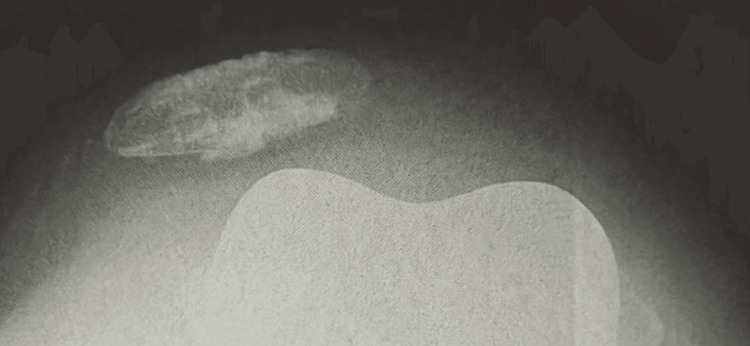
Postoperative skyline radiograph of a 67-year-old male suffering from repetitive patellar dislocations due to the internally rotated femoral component

Surgeons who choose not to replace the patella must accept that their patients will have the same or greater degree of prior knee pain and a significantly higher risk of reoperation (Figures 1-3).

Discussion

One of the most frequent problems following primary TKA is patellar resurfacing, which orthopedic surgeons who do total knee arthroplasty are still debating [37,38]. Concerns concerning patellofemoral resurfacing were exacerbated by the high incidence of postoperative anterior knee discomfort [39]. Although there is now more research comparing patellar resurfacing to non-resurfacing, the outcomes are still unreliable. Both patellar resurfacing and non-resurfacing are supported by the published literature; however, there is not enough convincing data to alter clinical practice.

Due to the danger of anterior knee discomfort and the potential for subsequent arthroplasty surgery, the decision not to replace the patella in a total knee arthroplasty might result in a low level of patient satisfaction. In addition, the decision not to replace the patella can take into account the lengthening of the procedure, financial concerns, the surgeon's level of experience, and the avoidance of complications such as fracture, osteonecrosis, component dissociation, wear, aseptic loosening, instability, overstuffing, and extensor mechanism rupture [40].

Patellofemoral overloading is common in patellas that have not yet been resurfaced, whether they have patella baja syndrome or not. In flexion, the distal patellar pole and the polyethylene inlay may come into contact [41]. Damage of the patellar tendon and iatrogenic joint line changes are the main causes of the unusually reduced length within the distal patellar pole and the tibial joint line after TKA [42,43].

Coory et al. [43] present the results from the Australian Orthopedic Association National Joint Replacement Registry of 570,735 primary knee arthroplasties that show a low rate of complications, surgical re-interventions, and a high degree of satisfaction of patients who have benefited from knee replacement with patellar resurfacing [5]. This is despite the fact that the design of knee prostheses is constantly changing, becoming more friendly from the point of view of patellar tracking [43-45].

## Conclusions

According to several research, individuals with total knee arthroplasty and non-resurfacing patella have better outcomes than those with complete knee arthroplasty with resurfacing patella from a functional standpoint. We include the potential for problems, the lengthened recovery period, the increased expense, and the inexperienced surgeons in some facilities as arguments against patellar resurfacing.

Patients who have had primary TKA suffer anterior knee pain regardless of whether the patella was resurfaced. Femoral internal rotation causes pain in the lateral border of the patella and lateral patellar tracking.

Patella baja is caused by excessive joint line elevation, which causes persistent overload and discomfort. The design of the TKR might have an impact on postoperative patellofemoral problems. After TKR, patellofemoral maltracking and patellar dislocation are often caused by surgical mistakes.

It is impossible to create a gold standard for total knee arthroplasty due to the absence of reliable comparative research on sizable patient populations and the low prevalence of anterior compartment discomfort in patients with total knee arthroplasty and non-resurfacing patella.
